# Cryo-EM structure of the Smc5/6 holo-complex

**DOI:** 10.1093/nar/gkac692

**Published:** 2022-08-22

**Authors:** Stephen T Hallett, Isabella Campbell Harry, Pascale Schellenberger, Lihong Zhou, Nora B Cronin, Jonathan Baxter, Thomas J Etheridge, Johanne M Murray, Antony W Oliver

**Affiliations:** Genome Damage and Stability Centre, School of Life Sciences, University of Sussex, Falmer, UK; Genome Damage and Stability Centre, School of Life Sciences, University of Sussex, Falmer, UK; Electron Microscopy Imaging Centre, School of Life Sciences, University of Sussex, Falmer, UK; Genome Damage and Stability Centre, School of Life Sciences, University of Sussex, Falmer, UK; London Consortium for CryoEM (LonCEM) Facility, The Francis Crick Institute, London, UK; Genome Damage and Stability Centre, School of Life Sciences, University of Sussex, Falmer, UK; Genome Damage and Stability Centre, School of Life Sciences, University of Sussex, Falmer, UK; Genome Damage and Stability Centre, School of Life Sciences, University of Sussex, Falmer, UK; Genome Damage and Stability Centre, School of Life Sciences, University of Sussex, Falmer, UK

## Abstract

The Smc5/6 complex plays an essential role in the resolution of recombination intermediates formed during mitosis or meiosis, or as a result of the cellular response to replication stress. It also functions as a restriction factor preventing viral replication. Here, we report the cryogenic EM (cryo-EM) structure of the six-subunit budding yeast Smc5/6 holo-complex, reconstituted from recombinant proteins expressed in insect cells – providing both an architectural overview of the entire complex and an understanding of how the Nse1/3/4 subcomplex binds to the hetero-dimeric SMC protein core. In addition, we demonstrate that a region within the head domain of Smc5, equivalent to the ‘W-loop’ of Smc4 or ‘F-loop’ of Smc1, mediates an important interaction with Nse1. Notably, mutations that alter the surface-charge profile of the region of Nse1 which accepts the Smc5-loop, lead to a slow-growth phenotype and a global reduction in the chromatin-associated fraction of the Smc5/6 complex, as judged by single molecule localisation microscopy experiments in live yeast. Moreover, when taken together, our data indicates functional equivalence between the structurally unrelated KITE and HAWK accessory subunits associated with SMC complexes.

## INTRODUCTION

The eukaryotic Structural Maintenance of Chromosomes (SMC) family includes the protein complexes cohesin, condensin and Smc5/6. At their respective ‘hearts’ sits an obligate heterodimer of two SMC proteins, either Smc1/Smc3, Smc2/Smc4 or Smc5/Smc6. Globular entities found at both the N- and C-termini of each SMC protein are brought together in space, to form a so-called ‘head’ domain that is capable of binding to, and turning over, ATP. The two halves of the ATPase are connected by a structural excursion known as the ‘arm’; formed from sequential alpha-helical regions that coalesce to form a highly extended anti-parallel coiled-coil. The arm is interrupted at its apex (most distant point from the head) by the ‘hinge’ domain; a region of SMC proteins responsible for hetero-dimerisation with their obligate binding partner. The binding, hydrolysis, and release of ATP by the two head domains (one from each SMC protein) provides a secondary, more transient and regulated dimerisation interface. The ‘core’ of each SMC complex is then elaborated through binding of additional ‘non-SMC’ protein subunits or ‘elements’, to provide the distinct functionalities required for their respective cellular functions. For more expansive reviews of the SMC-family, including their respective functions and subunit compositions, see (1–8).

All three complexes are required for the organisation and management of chromosome architecture and structure throughout the cell cycle. Cohesin has well described roles in sister chromatid cohesion and the organisation of the interphase chromosomes into topologically associated domains or TADs, whereas condensin is required to compact chromosomes at mitosis. The Smc5/6 complex has roles in the processes of DNA replication and DNA damage repair; the complex acting to suppress / prevent formation of inappropriate structures that can form during homologous recombination-mediated rescue of replication forks that have stalled (or collapsed) on encountering replication ‘road-blocks’ or obstacles.

Alterations to the coding sequence of human Nse2 (non-SMC-element; generally written as either Nse or NSMCE) have been linked to primordial dwarfism and insulin resistance (9), with changes in Nse3 linked to severe lung disease immunodeficiency and chromosome breakage syndrome (LICS, 10). Interestingly, Smc5/6 is also specifically targeted for ubiquitylation and degradation by the regulatory protein X of hepatitis B virus (HbX) to alleviate restriction of viral replication by the complex (11). The complex has also been shown to act as a restriction factor working against other viruses (12–14).

As well as containing subunits that provide both ubiquitin and SUMO E3-ligase activity (through Nse1 and Nse2 respectively), Smc5/6 is further differentiated from the cohesin and condensin complexes by the fact that the two non-SMC proteins (Nse1 and Nse3) that bind to its kleisin subunit (Nse4) belong to the ‘KITE’ family (kleisin-interacting tandem winged-helix element; 15) rather than the distinct and structurally unrelated ‘HAWK’ family (HEAT proteins associated with kleisins; 16). As proteins of the KITE-family are also found in prokaryotic SMC complexes, it has led to the hypothesis that Smc5/6 best represents the eukaryotic ‘cousin’ of *Bacillus subtilis* Smc/ScpAB and *Escherichia coli* MukBEF (15)—however, it is still unclear as to when in the evolutionary timescale KITEs were replaced by HAWK subunits, to create cohesin and condensin complexes.

Until relatively recently, structural information for components of the Smc5/6 complex has been limited to X-ray crystal structures for the ‘arm’ of budding yeast Smc5 in complex with Nse2 (PDB: 3HTK; 17), the complex between human Nse1 and Nse3 (3NW0; 18), and the isolated hinge-region of the fission yeast complex (5MG8; 19). However, recent additions have now expanded this to include an X-ray crystal structure of the *Xenopus laevis* Nse1/Nse/Nse4 hetero-trimer (7DG2; 20), cryo-EM and X-ray crystal structures for the budding yeast Nse5/6 hetero-dimer (PDB: 7LTO and 7OGG, respectively; 21,22), plus a complex of a Ubc9-SUMO mimetic bound to the Smc5-arm / Nse2 assembly (7P47; 23).

Here, we have determined a cryo-EM structure for the budding yeast Smc5/6 ‘holo-complex’, in its *apo* or non-liganded form. Our study serves to confirm the overall architecture of the complex, plus provide details of how and where the Nse1/Nse3/Nse4 KITE-kleisin subcomplex interacts with the Smc5/Smc6/Nse2 core. Additional experiments also reveal the presence of a crucial interface formed between the head domain of Smc5 and Nse1, which utilises the equivalent of the ‘W-loop’ or ‘F-loop’ found in Smc4 and Smc1 respectively (24). Taken together, our data uncover an unanticipated degree of functional equivalence between KITE and HAWK accessory subunits.

## MATERIALS AND METHODS

### Expression and purification

Detailed experimental procedures for both expression and purification of the *S. cerevisiae* Smc5/6 holo-complex are available in (25). For convenience, the composition of the two buffers that were used in this study are listed below:

**BUFFER C:** 20 mM HEPES.NaOH pH 7.5, 100 mM NaCl, 0.5 mM TCEP

**BUFFER F:** 20 mM HEPES.NaOH pH 7.5, 0.5 mM TCEP

### Cryo-EM

#### Sample preparation

Fractions eluting from a Superose 6 size exclusion chromatography column (equilibrated in BUFFER C; Cytiva Life Sciences, Little Chalfont, UK), corresponding to BS3-crosslinked holo-complex, were immediately used for grid preparation. The sample was diluted by a factor of two (with addition of BUFFER F) to reduce the overall NaCl concentration to 50 mM and yield a final concentration of 0.1 mg/ml. From this, 3 μl was applied to a freshly glow-discharged grid (Quantifoil R0.6/1 Cu 300 mesh grid; 60 s, 15 mA, PELCO easiGlow—Agar Scientific, Stansted, UK). Using an EM GP2 automatic plunge freezer (Leica Microsystems, Wetzlar, Germany) the grid was held in a chamber at 10°C and 90% relative humidity, for a period of 10 seconds, before blotting for 2.5–4.5 s using the auto-sensor. The grid was immediately plunged into liquid ethane at −182 ° and then stored under liquid nitrogen until data collection.

#### Data collection

Data were collected at LonCEM (The Frances Crick Institute, London, UK) at 300 kV on a Titan Krios electron microscope (Thermo Fisher Scientific, Waltham, MA USA), equipped with a Gatan K3 detector operating in counted super-resolution mode. Movies were acquired using EPU (Thermo Fisher Scientific). Data were collected from four grids across separate sessions, without hardware binning at a calibrated pixel size of 0.55 Å. Target defocus was −1 to −3.5 μm, with a total dose of 50 electrons per Å^2^, during an exposure time of 3.9–5 s, fractionated into 38–50 frames.

#### Data processing

An overview of the workflow used to process data is provided in Supplementary Figure S1. In summary, all movie frames were aligned using 5 × 5 patches in Motioncor2 with dose-weighting (26). All data were binned by a factor of 2. Contrast transfer function (CTF) parameters were estimated using CTFFIND4 (27). Particles were picked using Topaz (28) integrated into CryoSPARC. Initial training used a set of particles manually picked from a few micrographs, with the resultant ‘trained’ model being used to auto-pick particles across all micrographs. Apart from the ‘Head-only’ approach, all processing was carried out in parallel, using both CryoSPARC (v3.1.0, (29)) and RELION (v3.1, (30)) software suites, to yield similar maps. For the sake of simplicity and brevity, only maps with the highest resolution estimates are reported here. All refinements were performed using independent data half-sets (gold-standard refinement) and resolutions determined based on the Fourier shell correlation (FSC = 0.143) criterion (31).

#### Holo-complex

After several rounds of 2D classification in cryoSPARC, particles from ‘good’ classes were used for an initial *ab initio* reconstruction. The resultant particles were exported to Relion and then re-classified in 2D. Subsequent rounds of 3D classification in Relion yielded six classes, of which one (1: HOLO-COMPLEX; 17,152 particles, representing 13% of the input) provided the highest resolution after 3D refinement with a soft-edged mask and solvent flattening. Post-processing produced a map at 10.8 Å resolution.

#### Hinge/Arm

Working in parallel, using cryoSPARC, particle subtraction was used to remove the ‘head-end’ of the complex, before additional rounds of 3D classification, refinement and post-processing yielded a map at 8.53 Å (2: HINGE/ARM; 106 660 particles, 62.4% of input). A similar strategy was used to remove the ‘hinge-end’ of the complex, however further rounds of processing was halted due to the superior results obtained via the ‘head-only’ approach described below.

#### Head-only

A second trained ‘picking model’ was used to identify particles corresponding to just the ‘head-end’ of the complex. After several rounds of 2D classification, particles from ‘good’ classes were used for an initial *ab initio* reconstruction. After iterative rounds of 3D classification and refinement, one class was selected to take forward into non-uniform refinement, after removal of density corresponding to the HaloTag attached to the Nse4 subunit by particle subtraction (3: HEAD-ONLY; 84 810 particles, 51.7% of input). Post-processing yielded a map at 6.5 Å resolution.

#### Model building and generation of composite Map

An initial pseudo-atomic model for the Smc5/6 complex was generated by fitting of Phyre2-generated homology models into map segments (32), using programs of either the PHENIX software suite (33) or ChimeraX (33–35), plus additional manual positioning of subunits using either Coot (36) or PyMOL (the PyMOL Molecular Graphics System, Version 2.3.2, Schrödinger LLC). Please see Table 1 below for additional detail.

**Table 1.** Details of homology and AlphaFold models used in this study

		Phyre2 homology models — templates					*S. cerevisiae* AlphaFold model
Protein name	Model extent	PDB accession code	Protein	Species	Chain	Method^c^	Identifier
Nse1	Full-length	3NW0	Nse1	*H. sapiens*	A	X-ray	AF-Q07913-F1
Nse2	Full-length^a^	3HTK	Nse2	*S. cerevisiae*	C	X-ray	AF-P38632-F1
Nse3	Full-length	3NW0	Nse3	*H. sapiens*	B	X-ray	AF-Q05541-F1
Nse4	N-terminal domain^b^	6YVU	Brn1	*S. cerevisiae*	C	EM	AF-P43124-F1
Nse4	C-terminal domain^b^	6YVU	Brn1	*S. cerevisiae*	C	EM	AF-P43124-F1
Nse4	Middle section	7DG2	Nse4	*X. laevis*	D	X-ray	AF-P43124-F1
Smc5	Head (N-terminal)	5XEI	Smc	*P. yayanosii*	A	X-ray	-
Smc5	Head (C-terminal)	5XEI	Smc	*P. yayanosii*	A	X-ray	-
Smc6	Head (N-terminal)	5XEI	Smc	*P. yayanosii*	A	X-ray	-
Smc6	Head (C-terminal)	5XEI	Smc	*P. yayanosii*	A	X-ray	-
Smc5	Full-length	6YVU	Smc4	*S. cerevisiae*	B	EM	AF-Q08204-F1
Smc6	Full-length	6YVU	Smc2	*S. cerevisiae*	A	EM	AF-Q12749-F1

^a^Used directly as model.

^b^Generated by one-to-one threading (expert mode of Phyre2).

^c^X-ray = X-ray crystallography; EM = cryo-electron microscopy.

Two useful starting points for assembly of the model were achieved by placement of the Phyre2 homology model of Nse1 into the ‘head-only’ map (phenix.dock_in_map, CC [correlation coefficient] = 0.58; (33)) and the X-ray crystal structure of *S. cerevisiae* Nse2 in complex with a short, coiled coil section of Smc5 (PDB accession code: 3HTK; 17) into the ‘hinge-arm’ map (CC = 0.52).

The combine_focused_maps module of the Phenix software suite was subsequently used to generate a composite map, using each of the reported maps and the initial model as an alignment reference. This model was subsequently updated using the coordinates for each subunit generated by AlphaFold (AF) upon their public release. For the coiled coil ‘arms’ of both Smc5 and Smc6, it was necessary to first ‘break’ the AF-model into discrete sections, and then manually place these into the appropriate section of density; the individual AF-models are folded back upon themselves, adopting a more ‘globular’ conformation, rather than the expected linear / extended conformation. The overall fit and geometry of the final pseudo-atomic model was optimised using phenix.real_space_refine against the composite map (see Supplementary Table S1 for statistics relating to data collection, refinement, and model quality). Assembly of the final pseudo-atomic model was also informed by visual inspection and comparison to the cryo-EM structure of budding yeast condensin (PDB: 6YVU; 37). The final model contains the following amino acid ranges, with gaps in sequence arising from regions of either low sequence complexity/predicted disorder (AlphaFold) or where there was insufficient density in the composite map to reliably position or assign connecting loops: Smc5—amino acids 35–390, 394–882, 884–1068; Smc6—74–213, 215–290, 292–370, 387–431, 436–805, 814–919, 922–1104; Nse2—1–267; Nse1—11–336; Nse3—9–99, 114–152, 173–303; Nse4—40–124, 184–202, 213–245, 283–402. Given the overall resolution of our cryo-EM data, individual *B*-factor refinement was not used. Instead, atoms were manually assigned with a value of either 50.00 or 999.00, dependent on whether they corresponded to a section of the model with strong homology to existing structural data or not. For Nse4, an additional region of density compatible with an alpha helix was evident, however, as it was not possible to unambiguously assign this to a given amino acid sequence, it was modelled as containing only alanine/glycine residues and tentatively assigned as representing amino acids 182–202.

#### Data availability

The maps used to generate the composite cryo-EM volume have been deposited in the Electron Microscopy Data Bank (EMDB) with accession codes EMD-13893 (head-end of complex) and EMD-13894 (hinge and arm-region). Coordinates for the pseudo-atomic model of the Smc5/6 holo-complex have been deposited in the Protein Data Bank (PDB) with accession code PDB-7QCD. The accompanying composite cryo-EM volume has been deposited with accession code: EMD-13895.

#### Generation of yeast strains

Synthetic DNA encoding the genomic sequence for both WT and mutant versions of each gene were purchased as ‘Strings DNA Fragments’ from GeneArt (Thermo Fisher Scientific, UK). These were cloned into the vector pAW8-natMX6 (38), at the PaeI/SalI restriction sites, through Gibson assembly via short regions of homology included during synthesis. PCR was then used to amplify the gene and associated nourseothricin resistance module for introduction into the endogenous locus of diploid yeast cells, using lithium acetate transformation. Strains were generated from three individual haploid isolates, generated from tetrad dissections (see Table 2 for additional information).

**Table 2.** *Saccharomyces cerevisiae* strains used in this study

STRAIN TABLE		
Strain #	Genotype	Reference
2655	*MATalpha [leu2-3 trp1-1 ura3-1 can1-100 ADE2 his3-11,15]*	W303 (39)
2747 diploid	*MATa/alpha [leu2-3 trp1-1 ura3-1 can1-100 ade2-1/ADE2 his3-11,15 SMC5/smc5-Y961A, W964A::NATmx6]*	This study
2753 isolate 1	*MATa [leu2-3 trp1-1 ura3-1 can1-100 ADE2 his3-11,15 smc5-Y961A, W964A::NATmx6]*	This study
2755 isolate 2	*MATalpha [leu2-3 trp1-1 ura3-1 can1-100 ADE2 his3-11,15 smc5-Y961A, W964A::NATmx6]*	This study
2756 isolate 3	*MATalpha [leu2-3 trp1-1 ura3-1 can1-100 ADE2 his3-11,15 smc5-Y961A, W964A::NATmx6]*	This study
2745 diploid	*MATa/alpha [leu2-3 trp1-1 ura3-1 can1-100 ade2-1/ADE2 his3-11,15 SMC5/smc5-F972A, L978D, L981N::NATmx6]*	This study
2793 diploid	*MATa/alpha [leu2-3 trp1-1 ura3-1 can1-100 ade2-1/ADE2 his3-11,15 NSE1/nse1-F217A, E228R, R242A::NATmx6]*	This study
2794 isolate 1	*MATa [leu2-3 trp1-1 ura3-1 can1-100 ADE2 his3-11,15 nse1-F217A, E228R, R242A::NATmx6]*	This study
2795 isolate 2	*MATa [leu2-3 trp1-1 ura3-1 can1-100 ADE2 his3-11,15 nse1-F217A, E228R, R242A::NATmx6*	This study
2796 isolate 3	*MATalpha [leu2-3 trp1-1 ura3-1 can1-100 ADE2 his3-11,15 nse1-F217A, E228R, R242A::NATmx6]*	This study
2671	MATα *[ura3-1 his3-11,15 leu2-3,112 can1-100 ade2-1::ADE2-URA3 GFP-nic96::HIS3 nup170-GFP::URA3]*	SWY2049 (40)
2833	*MATalpha [ura3-1 his3-11,15 leu2-3,112 trp1-1 can1-100 ade2-1::ADE2-URA3 NSE4-Halo::hphMX6 GFP-nic96::HIS3 nup170-GFP::URA3]*	This study
2837	*MATalpha [ura3-1 his3-11,15 leu2-3,112 trp1-1 can1-100 ade2-1:ADE2-URA3 NSE4-Halo::hphMX6 GFP-nic96::HIS3 nup170-GFP::URA3 Nse1 F217A E228R R242A::NATmx6]*	This study

For single-molecule localisation microscopy, strains were generated by PCR amplification of the coding sequence for the HaloTag and nourseothricin resistance module from the plasmid pAW8-HaloTag-natMX6, using primers that also encoded regions of homology (100 bp) to the genomic regions flanking the C-terminus of Nse4. The resulting amplicon was used to transform diploid cells as before. Tetrad dissections produced nourseothricin resistant haploid cells, which were then crossed with strains expressing GFP-fusions of the nuclear pore components Nic96 and Nup170; enabling optimal focal planes to be determined for data collection (Table 2).

#### Design of mutations

Mutation sets were designed to disrupt localised structure or alter key features within a loop; typically, by reduction in the size of a given hydrophobic amino acid residue, and / or through alteration to an amino acid of a similar size that either neutralises or reverses charge.

### Single molecule localization microscopy (SMLM)

#### Sample preparation

Yeast were grown in synthetic complete media (SC-media; Formedium, UK) until they reached mid-log phase. JFX650 HaloTag ligand (Luke Lavis, Janelia Research Campus, Virginia, USA) was then added to the media to a final concentration of 50 nM. Cultures were grown for an additional period of 30 min, after which cells were harvested by centrifugation, transferred to fresh, non-supplemented media, where they were allowed to grow for a second incubation period of 20 min. Cells were again harvested by centrifugation, resuspended in 1ml of SC-media, then deposited onto an agarose pad (1% w/v agarose, 50% v/v OptiPrep, 50% v/v 2× SC-media). Samples were mounted on an ozone-cleaned circular coverslip (#1.5H, 25 mm; ThorLabs, New Jersey, USA) and placed in an Attofluor Cell Chamber for imaging (Thermo Fisher Scientific, UK).

#### Data acquisition

Live *S. cerevisiae* cells were imaged using a custom-built microscope; described previously in (41). Samples were mounted on a motorised microscope stage, kept at 30°C by use of a heated incubation chamber (Digital Pixel, UK). Both 488 nm (iBeam, Toptica, Munich, Germany) and 642 nm imaging lasers (Cube, Coherent, Inc., Daventry, UK) were expanded, collimated, and focused to the back focal plane of an apochromatic 1.45 NA, 60× TIRF objective (UIS2 APON 60× OTIRF, Olympus Europa, Hamburg, Germany). Illumination beams were angled in a highly inclined near-TIRF manner to achieve high signal-to-background. Fluorescence emission from the sample was filtered with either a 525–40 nm (GFP) or 692–40 nm (JFX650) band-pass filter (Semrock, IDEX Health and Science, New York, USA), expanded 2.5× and projected onto an Evolve 512 Delta EMCCD camera (Photometrics, Arizona, USA) with final image pixel size of 101 nm.

At the start of an experiment, cells were first illuminated using the 488 nm laser and a focal plane chosen from imaging the two GFP-labelled nuclear pore components (Nic96 and Nup170 proteins respectively; see Table 2). Continuous illumination was then provided by switching to the 642 nm laser (13 mW at rear aperture of objective lens) to provide a bleaching/excitation regime sufficient for single-molecule imaging. Movies consisting of 30 000 frames were recorded with an exposure time of 10 ms per frame. During preliminary experiments we found that a sub-population of JFX650 molecules could be photo-converted back from a long-lived dark-state when illuminated by a pulse of the 488 nm laser. We took advantage of this during data acquisition, controlling JFX650 emission such that the nuclei of each imaged yeast cells typically only contained a single emitting molecule at any one time. To ensure that single-molecule traces were recorded from a sufficient number of nuclei, each experimental repeat consisted of data collected across three separate fields of view, imaged one after the other.

#### Data analysis

Raw data were processed and analysed using a series of custom-written plugins for Fiji (https://imagej.net/software/fiji; (42)); collectively known as the ‘GDSC Single Molecule Light Microscopy Plugins—GDSC SMLM’. The plugins are freely available from both a Fiji update site (https://imagej.net/list-of-update-sites) and from GitHub (https://github.com/aherbert/GDSC-SMLM). Initial processing was carried out with the ‘PeakFit’ plugin, where fluorescence corresponding to single JFX650 molecules is localised by fitting of a 2D gaussian point-spread function; here, only signals with a minimum of 40 photons and localised with a precision of 45 nm or better were retained for downstream analysis. Molecular trajectories were then created from localisations appearing in consecutive movies frames, using the ‘Dynamic Multiple Target Tracing’ mode (43) of the ‘Trace Diffusion’ plugin. Parameters for this analysis were set as: diffusion coefficient = 0.6, temporal window = 5, local diffusion weight = 0.9, decay factor = 5, threshold = 0 frames, intensity model = false. For mean-square-displacement (MSD) analysis, the minimum trace length was set to five localizations (four displacements). Resulting values for apparent diffusion coefficients (*D*) were exported from Fiji, then plotted as frequency histograms, using log_10_ transformed values in GraphPad Prism (v. 9.3.1., GraphPad Software, San Diego, USA).

Molecular displacement data were also analysed using Spot-On (44), but with the minimum trace length parameter set at a value of 2 (1 displacement). Data were exported into a comma-separated value (.csv) file, using the GDSC SMLM ‘Trace Exporter’ plugin, before being uploaded to the Spot-On web interface (https://spoton.berkeley.edu). The following parameters were used for jump-length distribution analysis: bin width (μm) = 0.01, number of timepoints = 8, jumps to consider = 4, maximum jump (μm) = 2. Data sets were fit with a three-state model using the default parameters of Spot-On, with the exception of Dslowmin, localization fit error, and d*Z* (μm) which were set to 0.08, ‘yes’ and 0.9, respectively. Histograms corresponding to the calculated probability density function were plotted using the combined data from each of the three experimental repeats. Data were exported from Spot-On and plotted using Prism as before. Filled circles represent the value derived from each repeat, a solid bar their mean, and with error bars corresponding to 1 standard deviations (1 SD).

### Figures

Molecular images were generated using either PyMOL (v. 2.3.2) or ChimeraX (v. 1.1.1).

## RESULTS

In this manuscript, for simplicity and brevity, we refer to the protein components of the *Saccharomyces cerevisiae* SMC complexes, unless otherwise indicated.

**Figure 1.**
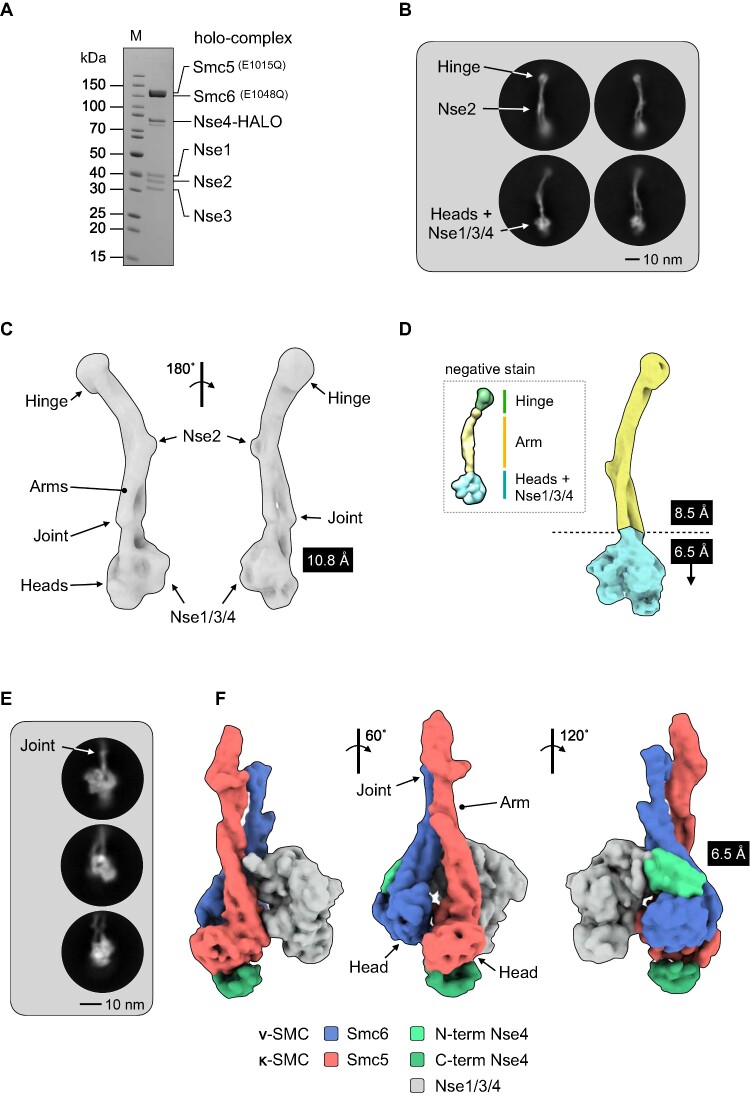
Cryo-EM of the budding yeast Smc5/6 complex. (**A**) Representative SDS-PAGE gel for the purified Smc5/6 holo-complex. (**B**) Representative 2D class averages (side views). Conformational flexibility leads to blurring of density at either the head (top) or hinge-end (bottom) of the complex. (**C**) Initial 3D map from cryo-EM at a resolution of 10.8 Å. (**D**) Maps from focussed refinement at 8.5Å and 6.5 Å for the indicated segments of the Smc5/6 holo-complex (d, inset) model obtained by uranyl acetate negative stain electron microscopy for comparison (25). (**E**) Representative 2D class averages (side views) and (**F**) resultant 3D map at 6.5 Å for the ‘head’-end of the complex. The cryo-EM map has been segmented and coloured with respect to its assigned component (see associated key for additional detail).

We have previously described reconstitution and characterisation of the *S. cerevisiae* Smc5/6 complex, using recombinant proteins expressed in insect cells (25). Here, cryo-EM data were collected for the six component ‘holo-complex’ stabilised by crosslinking with BS3, where Smc5 and Smc6 contain Walker B mutations E1015Q and E1048Q, respectively (Figure 1A, Materials and Methods).

A neural network was first trained with a set of manually picked particles (28). The resultant ‘picking model’ was then used to identify a total of 380 714 particles (see Supplementary Figure S1 for data-processing summary). Two sets of two-dimensional (2D) class averages emerged from processed data, with either the ‘hinge’ (Figure 1b, top) or the ‘head-end’ of the complex more clearly in focus (Figure 1B, bottom); the blurred density at either end consistent with a degree of conformational flexibility within the coiled coil ‘arms’ (25). From this, a sub-set of 17 162 particles were used to generate a medium-resolution 3-dimensional (3D) map that covered the entire length of the holo-complex, reconstructed at a resolution of 10.8 Å as judged by the 0.143 Fourier shell correlation (FCS) criterion (31) (Figure 1C). Focussed refinement allowed a slightly higher resolution map to be calculated for the upper (106 660 particles, 8.5 Å) segment of the complex (Figure 1D). Pleasingly, the resultant maps were consistent with the envelope we had previously obtained by uranyl acetate negative stain transmission electron microscopy ((25), Figure 1D, inset).

In parallel, we trained a second neural network with manually picked particles that encompassed just the head-end of the complex (824 644 particles). The resultant 2D class averages and 3D map from processed data (84 180 particles, 6.5 Å) are shown in Figure 1E and F, respectively. Segmentation analysis (34,45,46) allowed portions of the map to be readily assigned to the head domains, as well as the Nse1/Nse3/Nse4 (Nse1/3/4) subcomplex (coloured red, blue and grey respectively in Figure 1F). Additional segments of density associated with either the ‘head’ or ‘arm’ (dark or light green, Figure 1F) allowed the identity of each head-domain to be determined, due the expected binding positions of the N- and C-terminal domains of the kleisin Nse4 (see additional text below).

### A model for the Smc5/6 holo-complex

A sharpened composite map, generated from the highest resolution maps for each segment of the holo-complex, was of sufficient quality to allow positioning of the coiled-coil regions for both Smc5 and Smc6, as well as placement of the structure of *S. cerevisiae* Nse2 (Mms21) bound to the arm of Smc5 (PDB: 3HTK; (17)). This, combined with homology models for the ‘hinge’, ‘heads’ and Nse1/3/4, allowed an initial pseudo-atomic model to be constructed; subsequently updated using AlphaFold predictions made available through the EMBL-EBI repository (https://alphafold.ebi.ac.uk; (47)) (see Materials and Methods for expanded details).

**Figure 2.**
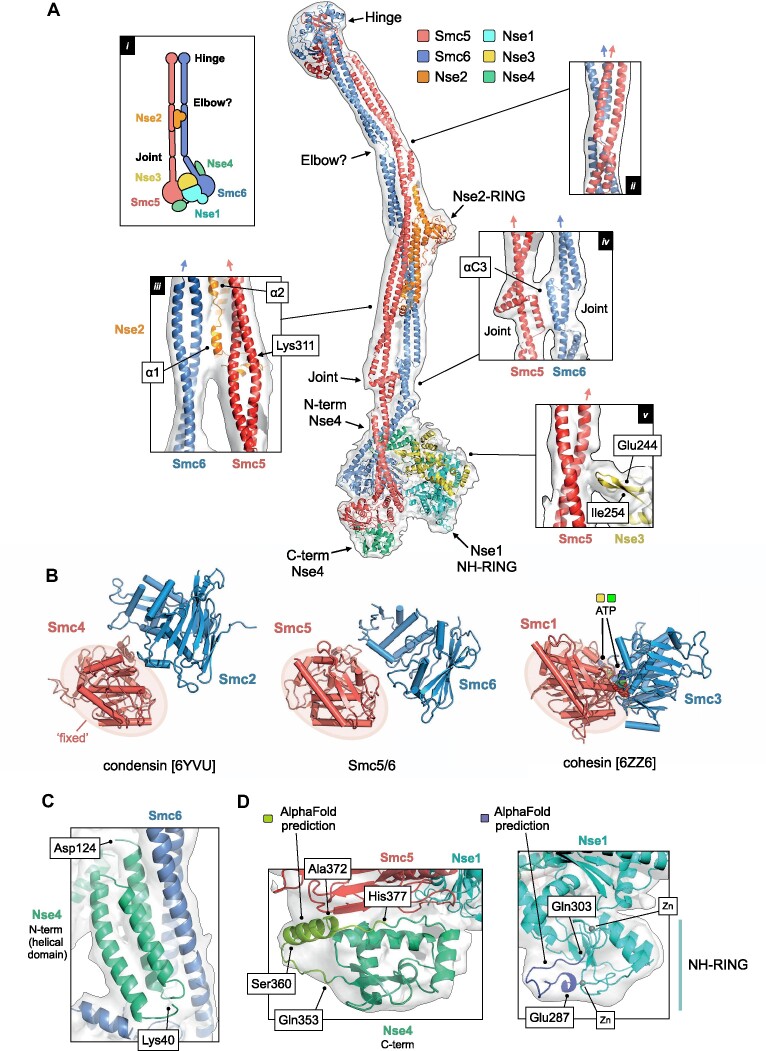
A pseudo-atomic model for the Smc5/6 holo-complex. (**A**) Overview of the pseudo-atomic model. (A, inset i) Schematic showing the overall architecture of the Smc5/6 complex and selected molecular features. (A, inset ii) Expanded view of the ‘Elbow’, highlighting the crossover of the coiled-coil ‘arms’ of Smc5 and Smc6 at this point. (A, inset iii) The first alpha-helix of Nse2 (α1) is situated between the two arms of the complex. The position of Lys311, a known site of auto-SUMOylation is also indicated (A, inset iv) Expanded view of the interface between the ‘Joint’ features of Smc5 and Smc6, involving the two αC3 helices. (A, inset v) A short beta-hairpin (amino acids Glu244-Ile254) protruding from Nse3 is in close proximity to the arm of Smc5. For each inset, the directionality of the ascending helix (head to hinge) is indicated by a blue or red arrow, for Smc5 and Smc6 respectively. (**B**) Comparison of the relative head domain positions in the cryo-EM structures of budding yeast condensin (PDB: 6YVU), Smc5/6 (this manuscript) and cohesin (PDB: 6ZZ6); in each, using the head of the κ-SMC as a fixed reference point. (**C**) Expanded view for the N-terminal helical domain of Nse4 (aa Lys40-Asp124) bound to the ‘arm’ of Smc6. (**D**, left) AlphaFold predicts the presence of an additional helical element (aa Ser360-Ala372) in the C-terminal domain of Nse4. (D, right). AlphaFold predicts a budding yeast-specific loop insertion in the NH-RING of Nse1 (aa Glu287-Gln303). Where shown, sections of density from the composite cryo-EM map are represented by a semi-transparent molecular surface, shaded in grey. Please also see associated key for additional detail.

Working from the ‘top’ of the complex downwards, we see that the hinge domain is tilted with respect to an axis defined by the coiled-coil arms; consistent with our previous analysis of this region in the fission yeast complex and with data published for condensin (19,48). Below this, there is an apparent discontinuity / break in the helices of the arms, located between the hinge and Nse2 subunit—a feature we believe represents the ‘elbow’ found in other complexes of the SMC-family ((37,49,50); see also Discussion). Notably, at this point the two arms of Smc5/6 also cross over each other (Figure 2A, inset ii).

In our previous lower-resolution study, we observed that the arms of Smc5/6 remain in close proximity (‘arms-together’, ‘rod-like’, ‘I’-conformation) for the majority of their length (25), in agreement with parallel studies published by other laboratories (21,22,51). With the additional resolution now afforded by cryo-EM, we see that the arms separate slightly from each other after the junction with Nse2 (Figure 2A, inset iii) but then remain approximately parallel until the head-end of the complex. Our model positions the first alpha-helix (α1) of Nse2 such that it can ‘talk’ across to the descending helix (hinge to head) of Smc6, as well as make its previously documented set of interactions with Smc5 (17); the helix appearing to ‘glue’ the two arms together, in agreement with crosslinking data for the holo-complex recently published by Taschener *et al.* ((22); Supplementary Figure S2a). The two arms then briefly re-contact each other, through the two inward facing helices (αC3) of the ‘joint’ (Figure 2A, inset iv)—a molecular feature common to SMC proteins (52) formed from three helix-loop repeats that encircle the more continuous ascending (head to hinge) alpha-helix, thus generating both an interruption and point of flexure within the coiled-coiled structure of the arm; again supported by crosslinking data (ref. (22); Supplementary Figure S2b;).

In our model, the head domains of Smc5 and Smc6 are not in direct contact. Whilst the resolution of our data is insufficient to directly observe the nucleotide status of the complex, a comparison of head domain positions indicates that our structure most resembles that of the apo/ATP-free/‘non-engaged’/juxtaposed/or ‘J’-state of budding yeast condensin deposited under PDB accession code 6YVU (37) rather than the ATP-bound/‘engaged’/‘E’-state seen for budding yeast cohesin when in complex with Scc2 and DNA (PDB: 6ZZ6; 53) (Figure 2B). Sections of the map corresponding to the RecA lobe of both the Smc5 and Smc6 head domain become less apparent at higher contour levels, suggesting a degree of conformational flexibility in this part of the complex, as supported by estimates of local resolution (Supplementary Figure S2c).

### Revealing the binding location of the Nse1/3/4 sub-complex

The Nse1/3/4 subcomplex is located to one side of the central axis defined by the coiled-coil arms of Smc5 and Smc6. The winged-helix 2 (WH/2) domain of Nse1 and the head domain of Smc5 are in direct contact, with a short loop protruding from the equivalent winged-helix domain of Nse3 (WH/B) positioned to interact with the arm of Smc5 (amino acids 244–254, Figure 2A, inset v). Notably, neither Nse1 nor Nse3 directly interact with Smc6.

The recent publication of an X-ray crystal structure for *Xenopus laevis* Nse1/3/4, has provided molecular details for how the kleisin subunit interacts with the Nse1/Nse3 KITE heterodimer (kleisin-interacting tandem winged-helix element). The central section of Nse4 follows a path through the centre of both KITE proteins, interacting with the linker regions that serve to connect their component winged-helix domains together (20). Pleasingly, our cryo-EM data allows integration of this kleisin path with the set of interactions made by the globular domains found at each of its termini: the helical N-terminal domain binding to the ‘neck’ of Smc6 (Figures 1F and 2C) and the C-terminal domain to the ‘cap’ of the Smc5 head domain (Figures 1F and 2D, left) thus confirming at the structural level the set of kleisin-facilitated interactions conserved across the SMC-family of complexes, including prokaryotic ScpAB and MukBEF as well as eukaryotic condensin and cohesin (52,54–60). Saying this, the resolution of our composite map is not sufficient to allow an unambiguous tracing of the parts of Nse4 that serve to connect the N-terminal domain to the central section (amino acids 125–183) or the central section to the C-terminal domain (aa 246–283), indicating either intrinsic disorder or a high degree of conformational flexibility in the *apo-*state; an observation consistent with the kleisin moieties in cryo-EM structures of other eukaryotic SMC complexes (37,53,61,62).

### Additional structural features are predicted by AlphaFold

AlphaFold predicts (albeit with a lower level of confidence) the presence of a hereto unknown alpha-helical element in the C-terminal domain of Nse4 (amino acids Ser360 through to Ala372). Nicely, this provides a facile explanation for a region of additional density visible in our composite map, not accounted for by the initial Phyre2-generated model (Figure 2D, left). Similarly, AlphaFold predicts the presence of a budding yeast-specific extended loop within the NH-RING domain of Nse1 (amino acids Glu287 to Gln303), which again provides a better overall fit to the experimental map (Figure 2d, right, Supplementary Figure 2d).

### A conserved loop in Smc5 with functional equivalence to the W- / F-loop

**Figure 3.**
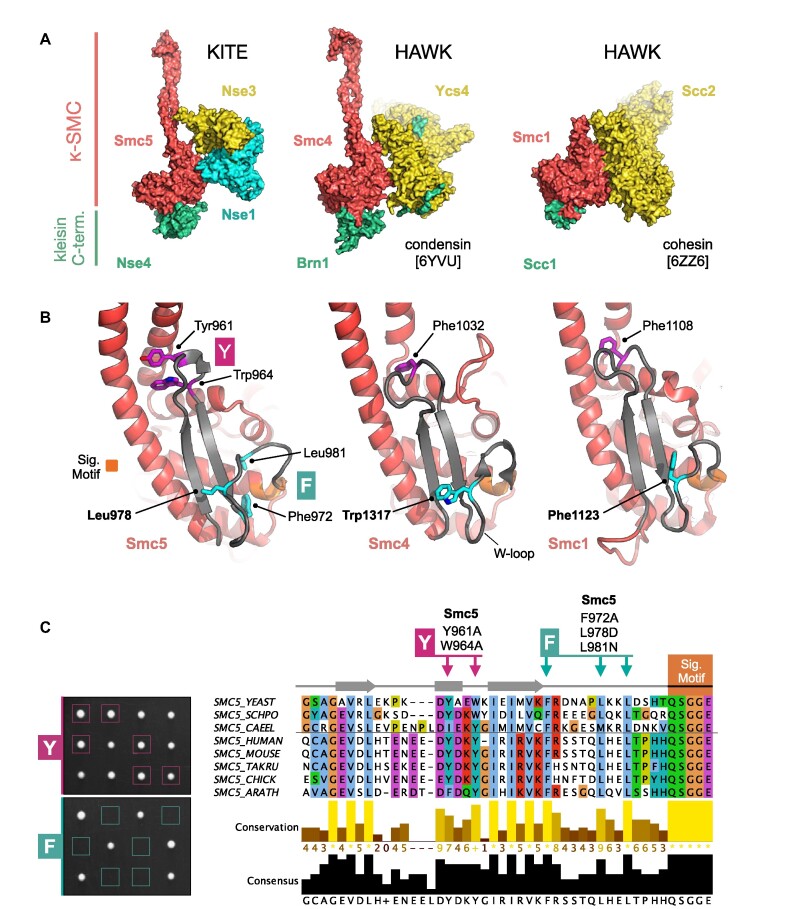
KITES and HAWKS share a common interaction interface involving the κ-SMC ‘W-loop’. (**A**) Side-by-side visualisation of the κ-SMC head domain from Smc5/6 (left), condensin (middle) and cohesin (right) in complex with their respective kleisin C-terminal domain; Nse4, Brn1 and Scc1. In each case, the interacting partner, whether KITE or HAWK, makes a similar set of interactions with the head domain of the κ-SMC. (**B**) Expanded view, showing secondary structure molecular cartoons for each κ-SMC head domain, highlighting the position of conserved amino acids within the ‘W-loop’ or equivalent (stick representation, carbon atoms coloured cyan) plus aromatic residues within the preceding sequence (stick representation, carbon atoms coloured magenta). The ABC-signature motif is additionally highlighted in orange. (**C**, left) Tetrad dissections. Spores derived from diploid *S. cerevisiae* strains carrying both wild-type allele and indicated mutant allele plus associated NAT-selectable marker (natMX6). Genotypes were confirmed by replica plating of spores on selective media (not shown). (C, right) Multiple sequence alignment, across selected species, showing conservation and consensus of amino acids within the W-loop and preceding region of Smc5 (produced using Jalview 2 with Clustal X colour scheme; (73)). Sets of compound mutations introduced into budding yeast: Smc5-Y = Y961A, W964A; Smc5-F = F972A, L978D, L981N. Please also see associated key for additional detail.

The location of Nse1/3/4 binding is particularly striking, as it echoes that of the structurally unrelated HAWK accessory proteins (HEAT proteins associated with kleisins) found in the condensin and cohesin complexes; in each case, the head domain of the κ-SMC (Smc5, Smc4, Smc1) serving to provide a major interaction surface (Figure 3A).

Closer inspection reveals that a region of Smc5 (amino acids Gly947—Gly978), which ‘talks’ across to the Nse1 subunit, is structurally equivalent to the ‘W-loop’ of the Smc4 head domain 24 (Figure 3B, C, inset). Notably, defined mutations (S1316D or W1317A) introduced into the W-loop render budding yeast cells non-viable. Furthermore, in the context of a fully recombinant ‘head complex’, mutation of an equivalent tryptophan residue in *Chaetomium thermophilum* (*Ct*) Smc4 resulted in a dramatic reduction of its ability to turnover ATP (24). In Smc1, the equivalent region has been given the alternative title of ‘F-loop’, due to the presence of a conserved phenylalanine residue (F1123 in budding yeast Smc1 (24,62,63).

In Smc5, Leu978 appears to be the structurally equivalent ‘F’ or ‘W-loop’ residue (Figure 3B)—a relationship supported by a strong preference for a leucine in this position, as revealed by a cross-species multiple amino acid sequence alignment (Figure 3C). However, two additional amino acids within the same loop, Phe972 and Leu981, are also strictly conserved (label F in Figure 3B and C). The same analysis identified a potential second region of conservation in the preceding sequence (labelled Y in Figure 3B and C). Here, structural comparison suggested that one or more aromatic residues might potentially act to anchor or connect the helical lobe of the head domain back to its coiled-coil arm. Interestingly, in *E. coli* MukB, this region is highly elaborated to form the so-called ‘larynx’, residues of which contribute to its DNA-binding interface (64).

When working with homology models and structural data of limited resolution, there is a degree of uncertainty as to the precise amino acids involved in a molecular interface. With this in mind, we introduced sets of mutations designed to either disrupt localised structure or alter key features within a loop (see Materials and Methods). Yeast harbouring the (Y) Y961A, W964A double mutation were viable and surprisingly displayed no sensitivity to a range of genotoxic agents. In sharp contrast, introduction of the (F) F972A, L978D, L981N triple mutant rendered budding yeast cells inviable, as determined by dissecting sporulated diploids; confirming the importance of this loop to cellular function (Figure 3C, Supplementary Figure S3a).

**Figure 4.**
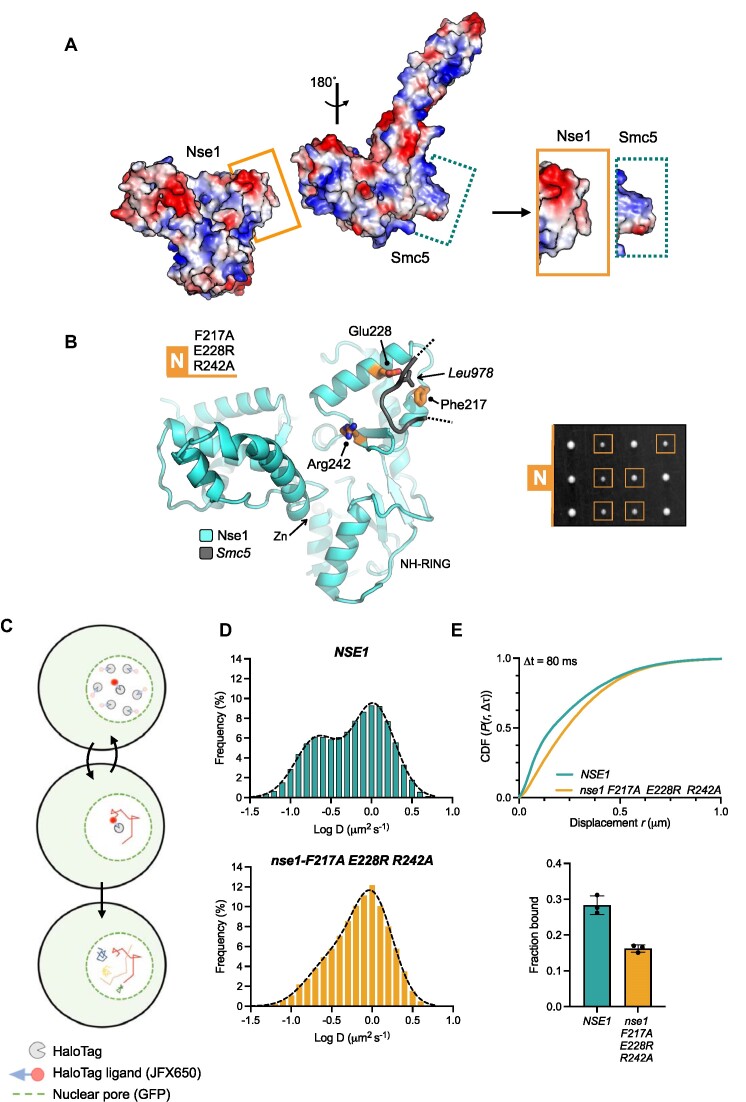
Examining the cellular effects of breaking the Nse1 / Smc5-loop interface. (**A**) Molecular surface representations for Nse1 and the head domain of Smc5, coloured by electrostatic potential (APBS plugin, PyMOL). The surface of Nse1 and the conserved loop extending from Smc5 display a high degree of charge complimentary (visualisation aided by rotation of the head domain through 180°. The interacting regions, as bounded by the drawn rectangles, are shown in an expanded view on the right. (**B**, left) Molecular secondary structure cartoon representation, showing the relative locations of the amino acids mutated within the Nse1/Smc5-loop interface: Nse1-N = F217A, E228R, R242A (b, right) Tetrad dissection. Spores derived from diploid *S. cerevisiae* strains carrying both wild-type and indicated mutant allele plus associated NAT-selectable marker (natMX6). Genotypes were confirmed by replica plating of spores on selective media (not shown). (**C**) Schematic of our single particle tracking approach. Yeast endogenously expressing Nse4 fused to a C-terminal HaloTag are labelled with JFX650 dye. Fluorescence from individual dye molecules is then used to calculate trajectories that represent the diffusion behaviour of the Smc5/6 complex containing the HaloTag fused Nse4 subunit. (**D**) Diffusion coefficient frequency histograms calculated using pooled data (three independent experimental repeats) in either the *NSE1* or *nse1-F217A E228R R242A* genetic backgrounds. (**E**, top), Cumulative distribution frequency plot of pooled single-molecule displacements. (E, bottom) ‘Fraction bound’ values determined from fitting of experimental data with kinetic models available within Spot-On. Filled circles represent the value determined from each technical repeat, with the height of the bar corresponding to the mean. Error bars represent one standard deviation. Please also see associated keys for additional details.

In contrast, the design of mutations aiming to disrupt the Nse1/Smc5-loop interaction was complicated by the lack of strong amino acid sequence conservation in cross-species alignments (data not shown). However, calculation of electrostatic surface potentials revealed a high degree of charge-complementary between the two interacting surfaces (Figure 4A). Furthermore, comparison of the equivalent interface in the available AlphaFold models (budding yeast, fission yeast and human) suggests co-evolution of the two surfaces, and thus by extension, a selective pressure to conserve the interaction (Supplementary Figure S4). With this in mind, we introduced the ‘N’-set of mutations into yeast, designed to alter the charge/hydrophobicity properties of the Nse1 surface (N: F217A, E228R, R242A; Figure 4B, left). Here, yeast were viable, but displayed a slow growth phenotype and mild sensitivity to treatment with MMS or camptothecin (Figure 4B, right; Supplementary Figure S4).

### Live-cell single-particle tracking of Smc5/6 in budding yeast

We have shown previously that single-particle tracking (SPT) is an effective tool for quantifying the levels of Smc5/6 associated with chromatin in live fission yeast (41). To observe whether the N-set phenotype was generated by altered interaction with chromatin, we adapted and optimised our existing methodology so that similar experiments could be carried out in budding yeast.

We therefore generated a *S. cerevisiae* strain that endogenously expresses Nse4 fused to a HaloTag at its C-terminus, to facilitate specific fluorescent-labelling by the dye JFX650 (Materials and Methods, Figure 4C). Labelled, asynchronous, mid-log phase yeast cells were imaged at high temporal resolution (10 ms exposure, 100 Hz) on a custom-built microscope (Materials and Methods). Nse4-HaloTag-JFX650 moieties were localized, and their movements traced to create a dataset of single-molecule trajectories in both wild-type (*NSE1*) and mutant (*nse1-*F217A E228R R242A) genetic backgrounds. Apparent diffusion coefficients (*D*) derived from mean-squared-displacement (MSD) analyses identified a bi-modal distribution in the *NSE1* background; suggesting the presence of at least two sub-populations of molecules, either fast, freely diffusing (*D* = ∼1 μm^2^/s) or slower, chromatin-interacting (*D* ≤ 0.2 μm^2^/s; Figure 4D, top). In contrast, the equivalent analysis of the mutant strain revealed a strong bias towards a more freely diffusing population (Figure 4D, bottom). As in our previous work, we also used the bias-aware kinetic modelling software ‘Spot-On’ to extract apparent sub-populations from our SPT data (44). Here, in agreement with the MSD-derived data, a cumulative distribution frequency plot (CDF) also showed a decrease in small range-displacements for the mutant strain (Figure 4E). Fitting of the data with the kinetical models available in Spot-On, indicated an overall decrease in the static / chromatin-bound fraction of the Smc5/6 complex from around 27% (of the total) in the ‘wild-type’ *NSE1* background to around 15% in the mutant background.

Together, these data illustrate that the electrostatic interaction between the surface of Nse1 and the loop protruding from Smc5 plays an important role in ensuring proper chromatin association/retention of the Smc5/6 complex.

## DISCUSSION

Using recombinant proteins expressed in insect cells, we have reconstituted and then visualised by cryo-EM the six-subunit budding yeast Smc5/6 ‘holo-complex’ in its *apo* or unliganded form—to provide an overview of the complex's architecture and reveal the position of the bound Nse1/3/4 sub-complex. Whilst structurally unrelated to the HAWK-family of proteins (HEAT proteins associated with kleisins; (16)) the set and type of interactions made by the KITE (kleisin-interacting tandem winged-helix element; (15)) hetero-dimer of Nse1/3 indicates a high degree of functional equivalence between the two different types of kleisin-associated subunit.

### Functional conservation of a loop located in the κ-SMC subunit

A short loop, located in the head domain of the κ-SMC subunit, represents a structural feature found initially in both condensin and cohesin (W-loop and F-loop, respectively; (24)), and now confirmed here as also present in Smc5/6. Notably, where tested, non-conservative changes to amino acid residues within the loop are incompatible with the viability of yeast, acting to confirm both the importance and relevance of this relatively small molecular feature to the cellular function of eukaryotic SMC-complexes ((24), and this manuscript).

In their 2019 paper, Hassler *et al.* demonstrated that the W-loop of Smc4 (κ-SMC) was important to the ATPase activity of condensin, its stable association with chromatin, and for its interaction with the Ycs4 (HAWK) subunit. This led them to propose a model, where a conformational change in the head domain of Smc4, promoted by binding to ATP, leads to rotation of the two component sub-domains (helical- and RecA-lobe) with respect to each other, along with concomitant remodelling of the W-loop; such that it is now released from its *apo*-state interaction with Ycs4 and is no longer inhibitory to association of the Smc2 head domain (ν-SMC). Their follow-on study expanded upon this initial hypothesis to reveal a more complex ‘flip-flop’ mechanism, which involves a physical switch of HEAT subunit, from Ycs4 (binding to the κ-SMC head domain) in the apo/unliganded state, to Ycs1 (binding to the ν-SMC head domain) in the engaged/ATP-bound state (37).

We speculate here that a similar mechanism is at play in Smc5/6. A hypothesis supported by the need for a conformational change, to move from the *apo*- or non-engaged state visualised in this manuscript, to the ATP-bound state where the head-domain of Smc5 is fully engaged with that of Smc6. We believe that is accomplished by first breaking the Smc5 (κ-SMC)/Nse1 (KITE) interface, in a manner like condensin. By analogy, this proposes a similar ‘flip-flop’ mechanism, where ATP-binding to Smc5 would serve to first release Nse1, such that Nse3 is unconstrained and now able to interact with Smc6 to allow full head-engagement. Another parallel with the behaviour of condensin adds credence to this idea, as disruption of the Smc5/Nse1 interface also results in a global reduction in the amount of Smc5/6 associated with chromatin, as demonstrated by our single molecule tracking experiments. Moreover, since the C-terminal HEAT repeat of Scc2 (HAWK) binds to the F-loop of Smc1 (κ-SMC) in cohesin and is reported to make no contact with Smc3 (ν-SMC) in its *apo*-state (63), it strongly hints that the ‘flip-flop’ mechanism may represent a conserved, sequential set of steps that underpin activation of cohesin, condensin and Smc5/6 (see additional discussion below).

### Comparison of the MukBEF and Smc5/6 apo-states

Like Smc5/6, the homo-dimeric SMC complexes of enterobacteria and γ-proteobacteria, Smc/ScpAB and MukBEF respectively, also contain KITE rather than HAWK subunits (15). As the series of cryo-EM structures reported for the *Photorhabdus thracensis* MukBEF complex also include that of an *apo-*state (PDB:7NNY; ref. 64) it serves as a useful comparator.

**Figure 5.**
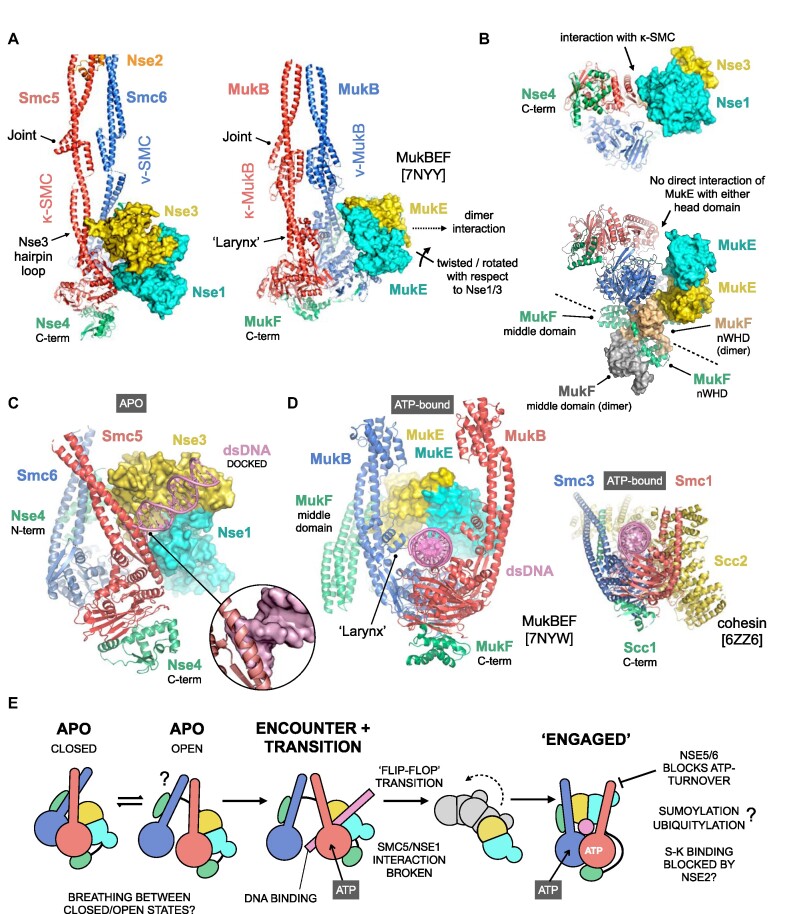
Exploration of conformational changes likely to accompany binding of dsDNA/ATP. (**A**) Comparison of the *apo*-states for Smc5/6 and the related MukBEF complex (PDB: 7NYY). To aid visualisation, only subunits with direct equivalence across both complexes are shown, and for clarity molecular surfaces instead of secondary structure cartoons are shown for the respective KITE proteins. A direct contact between the ‘joint’ features of Smc5 and Smc6 serves to generates a fully ‘closed’ conformation similar to that reported for MukBEF (64) but without a reinforcing secondary ‘larynx’ interface. Furthermore, the KITE homodimer formed by MukE sits, and is held, in a different position when compared to the KITE heterodimer of Nse1/Nse3, largely as a result of its more elaborate interaction with its dimeric kleisin partner MukF. (**B**) In the *apo-*state, the MukE heterodimer makes no direct interaction with the head domain of either MukB protein, instead making a series of interactions with the domain-swapped N-terminal winged-helix domain (nWHD) of MukF that anchor it in place. (**C**) Simple superposition of a DNA duplex, taken from the docking pose reported for the human NSE1/3 heterodimer (65) onto our *apo-*state structure, indicates that without accompanying conformational changes extension of the trajectory for the bound DNA would generate steric clashes with the arm of Smc5 (inset, DNA now shown in surface representation). (**D**) DNA/ATP-bound forms of MukBEF (left) and cohesin (inset, right), providing side-by-side comparisons and a visualisation aid of the expected fully ‘engaged’ conformation of SMC-complexes. (**E**) A speculative model for how the Smc5/6 complex might bind to and engage with dsDNA. We propose that the *apo-*state can ‘breathe’ between a fully closed conformation and a more open state, which allows / facilitates binding of dsDNA to the positively charged surface / groove created at the interface of Nse1/Nse3, to generate an intermediary ‘encounter’ complex. This, along with concomitant binding of ATP to the head domain of Smc5, serves to break the Smc5/Nse1 interaction allowing a ‘flip-flop’-type transition to the anticipated fully ‘engaged’ state. It is not clear how, or indeed if, ubiquitylation, SUMOylation or other post-translational modification affects either conformation or ATPase activity. It is also not known if the presence of Nse2, acts to block binding or transition of bound dsDNA into the S-K ring (SMC-kleisin) compartment. Binding of the Nse5/6 heterodimer blocks the ability of Smc5/6 to turn over ATP (22,25), but it is not fully known what effect this has on the overall conformation at the head-end of the complex.

A direct contact between the ‘joints’ from Smc5 and Smc6 serves to create a fully ‘closed’ conformation similar to that reported for MukBEF (Figure 2, inset iv and Figure 5A; (64)), albeit without the secondary reinforcing interface generated by association of the MukB ‘larynx’—which represents a molecular adaptation/feature not present in most other SMC proteins (64). Interestingly, the position of the short beta-hairpin protruding from Nse3, suggests that it might help stabilise the *apo-*state of Smc5/6, in lieu of a *bona fide* larynx, but this will of course require confirmation by experiment (Figure 2, inset v and Figure 5A). However, unlike Nse1/3 (or indeed the HAWK proteins Ycs4 and Scc2) the MukE homodimer does not contact the head domain of either SMC subunit. Instead, it is held in a more ‘dissociated’ state, as a consequence of its more elaborate interaction with the kleisin subunit (MukF); in particular that of the domain-swapped nWHD (N-terminal winged-helix domain) from its dimeric partner, which interestingly also serves to connect and anchor it to the head domain of ν-MukB (Figure 5B). Dimerisation of the kleisin moiety is another feature apparently unique to the MukBEF complex (64).

### A speculative model for DNA-binding and engagement

In Zabrady *et al.* (65), we reported a docking pose for a DNA duplex bound to the positively charged cleft formed between human Nse1 and Nse3 (NSMCE1/NSMCE3), guided by a range of supporting biochemical and biophysical data. A study using *Xenopus laevis* Nse1/3/4 has reported a similar mode of interaction (20). However, superposition of this docked pose onto our *apo-*state structure (Figure 5C) reveals that the DNA duplex (or extension of it, to represent a more physiological substate) directly clashes with the arm of Smc5 (Figure 5C, inset). Whilst the DNA could theoretically be accommodated via minor adjustments of the position of either the Nse1/3/4 subcomplex or head domain of Smc5, the resulting configuration would still be at odds with the expected position, i.e. sitting on top of the head domains, as illustrated here by comparison to the DNA-bound MukBEF complex (Figure 5D, PDB: 7NYW; (64)) and *S. cerevisiae* cohesin complex (Figure 5D, inset, PDB: 6ZZ6; (53)) but also observed for other proteins of the SMC-family, including the more distantly related Rad50 (66).

Interestingly in the MukBEF *apo-*state, the orientation of the MukE homodimer with respect to the two MukB promoters suggests that it may already be ‘primed’ to interact with dsDNA. Indeed, superposition of the *apo-* and DNA-bounds states of MukBEF (using ν-MukB as a fixed reference point) indicates that ν-MukB/MukE largely behaves as a single rigid body, already able to accept a DNA duplex, thus potentially removing the need for any prior conformational changes driven by the aforementioned ‘flip-flop’ mechanism (Supplementary Figure S5a).

Synthesising these findings, we propose a speculative model for how Smc5/6 might engage with and bind dsDNA (as illustrated by the schematic in Figure 5E). In the *apo*-state, the head-end of the complex can ‘breathe’ between closed and more open states, such that an initial ‘encounter’ complex can be formed between the Nse3 (KITE) subunit and dsDNA. We suggest that this event, along with concomitant binding of ATP to the head domain of Smc5, serves to break the Smc5/Nse1 interaction to allow a ‘flip-flop’ transition, which then generates the anticipated fully ‘engaged’ state. We also speculate here that association of the Nse5/6 heterodimer (or its functional human equivalent) may serve to preserve or trap this state (22,25). It is also not clear what effect, if any, auto-modification of the Smc5/6 complex though ubiquitylation and/or SUMOylation might have on its conformational state, in particular that of the head-end of the complex; these are open questions, that will require additional experimentation to answer.

### Does Nse2 prevent or control bending at the elbow?

We and others have observed some degree of flexure at the ‘elbow’ of Smc5/6 (21,22,25,51,67) but without the acute bend observed in condensin, cohesin and MukBEF (Supplementary Figure S5b; (37,49,50,53,61,62,68)). Notably, discontinuities in the ascending helix of Smc5 and in both helices of Smc6, as predicted by the current iteration of AlphaFold models (very low per-residue confidence scores, suggesting that they are unstructured) coincide with the point of flexure and a ‘kink’ in the trajectory of the fully associated arms (Figure 2A, labelled as ‘Elbow?’). However, the set of interactions made by Nse2 with Smc5, and now across to Smc6, may provide some insight, as these are likely to prevent separation and rotation of the arms, and therefore the concerted set of motions thought to produce a bend (49).

Interestingly, a major hotspot for auto-SUMOylation has been mapped to a region within the coiled-coil arm of Smc5, sitting between amino acids Lys310 and Lys327. Whilst the precise site(s) of modification appears to be redundant, Lys311 has been identified with high confidence as a major site of auto-SUMOylation (69). Saying this, it is not known what effect SUMOylation has on the overall structure/conformation of the Smc5/6 holo-complex, but the proximity of the primary modification site to the α1-helix of Nse2 hints at a regulatory role; potentially one that acts to control arm ‘architecture’ (Figure 2, inset iii) as well as performing the documented roles in promoting interactions with, and/or modulating activity of, replication fork associated-DNA helicases such as Sgs1^BLM^ (70) and Mph1^FANCM^ (71).

Interestingly, SUMOylation catalysed by the Smc5/6 complex has been shown to be stimulated by the addition of single-stranded DNA (ssDNA) to reaction mixes (72) with circular dichroism experiments showing localised changes in the secondary structure of Smc5 upon DNA-engagement. We speculate that this is likely to occur as a direct consequence of an initial binding event at the hinge-region of the complex, that leads to stimulation and auto-modification of Smc5/6, which then allows the complex to control/modulate homologous recombination activity at a stalled replication fork, presumably through regulation of specific helicases and/or other modified substrates; supported by our experimental observations in fission yeast, where mutations affecting the ability of the hinge to bind ssDNA lead to gross chromosomal rearrangements, but do not impact chromatin-binding or retention of the complex (41).

Finally, our cryo-EM structure of the apo/non-liganded state provides a highly valuable ‘stepping stone’ along the way to a fuller understanding of the set of molecular interactions and dynamics than underpin the cellular function(s) of the Smc5/6 complex. Clearly, a desirable goal is now the determination of structures for the ATP-bound and DNA-engaged states.

We note that during the review and revision process for this paper, a manuscript describing the cryo-EM structure for the ‘head-end’ of *S. cerevisiae* Smc5/6 in complex with ATP and dsDNA was published in Proceedings of the National Academy of Science (74).

## DATA AVAILABILITY

The two maps used to generate the composite cryo-EM volume have been deposited in the Electron Microscopy Data Bank (EMDB) with accession codes EMD-13893 (head-end of complex) and EMD-13894 (hinge and arm-region). Real-space refined coordinates for the Smc5/6 model have been deposited in the Protein Data Bank (PDB) with accession code PDB-7QCD. The accompanying composite cryo-EM volume has been deposited with accession code: EMD-13895.

## Supplementary Material

gkac692_Supplemental_File
