# Supplementary material for: Cryo-EM structure of the Smc5/6 holo-complex
Source: Nucleic Acids Res. 2022 Aug 22;50(16):9505–20. doi: 10.1093/nar/gkac692 (PMC9458440; doi:10.1093/nar/gkac692)
Supplement: gkac692_Supplemental_File [file gkac692_supplemental_file.pdf]

Supplementary Table 1 | Cryo-EM data collection, refinement, and validation statistics

	**(1) Head-only (EMD-13893)**	**(2) Hinge/Arm (EMD-13894)**	**(3) Composite (EMD-13895, PDB 7QCD)[1]**
**Data collection and processing**			
Magnification	88,000	88,000	88,000
Voltage (kV)	300	300	300
Electron exposure (e-/$Å^2$)	50	50	50
Defocus range (μm)	-1 to -3.5	-1 to -3.5	-1 to -3.5
Pixel size (Å)	1.1	1.1	1.1
Symmetry imposed	C1	C1	C1
Initial particle images (no.)	824,644	380,714	-
Final particle images (no.)	84,810	106,660	-
Map resolution (Å)	6.5	8.5	-
FSC threshold	0.143	0.143	-
**Refinement**			
Non-hydrogen atoms	-	-	25133
Protein residues	-	-	3137
B-factors ($Å^2$)	-		
Protein	-	-	not refined[3]
R.m.s. deviations	-		
Bond lengths (Å)	-	-	0.005
Bond angles (˚)	-	-	0.779
**Validation**	-		
MolProbity Score	-	-	3.11
Clashscore	-	-	33.06
Poor rotamers (%)	-	-	9.48
Ramachandran plot	-		
Favoured (%)	-	-	94.67
Allowed (%)	-	-	5.17
Disallowed (%)	-	-	0.16

[1] A pseudo-atomic model of the entire complex was built and refined against a composite map generated from map (1) and map (2)

[2] Values obtained from Local Resolution Map (Phenix)

[3] Individual B-factors were not refined

SUP. FIGURE 1

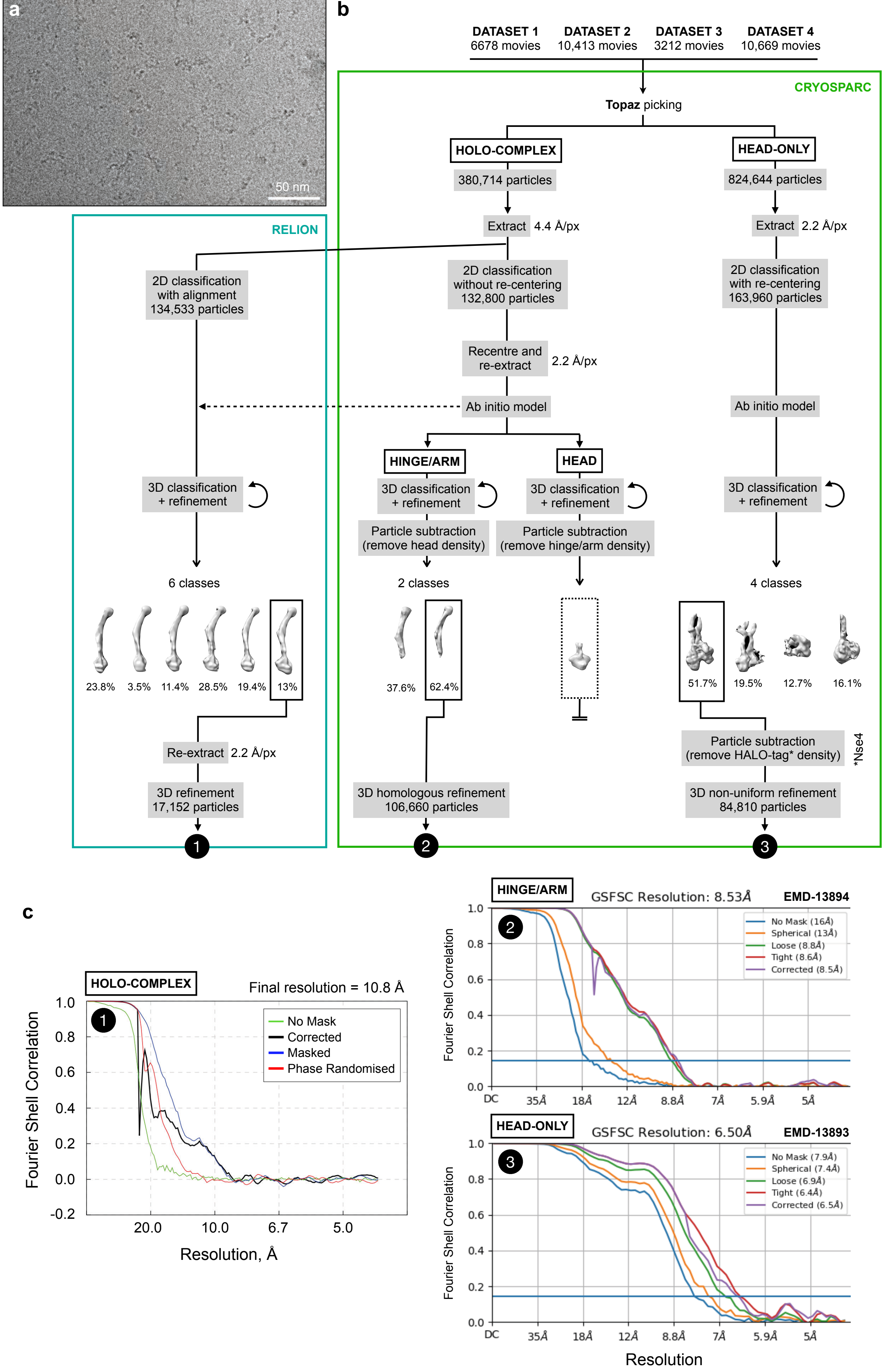
a
50 nm
b
DATASET 1
6678 movies
DATASET 2
10,413 movies
DATASET 3
3212 movies
DATASET 4
10,669 movies
CRYOSPARC
Topaz picking
HOLO-COMPLEX
380,714 particles
Extract 4.4 Å/px
2D classification without re-centering 132,800 particles
Recentre and re-extract 2.2 Å/px
Ab initio model
HINGE/ARM
3D classification + refinement
Particle subtraction (remove head density)
2 classes
37.6%
62.4%
3D homologous refinement 106,660 particles
HEAD
3D classification + refinement
Particle subtraction (remove hinge/arm density)
HEAD-ONLY
824,644 particles
Extract 2.2 Å/px
2D classification with re-centering 163,960 particles
Ab initio model
3D classification + refinement
4 classes
51.7%
19.5%
12.7%
16.1%
Particle subtraction (remove HALO-tag* density)
*Nse4
3D non-uniform refinement 84,810 particles
RELION
2D classification with alignment 134,533 particles
3D classification + refinement
6 classes
23.8%
3.5%
11.4%
28.5%
19.4%
13%
Re-extract 2.2 Å/px
3D refinement 17,152 particles
1
2
3
c
HOLO-COMPLEX
Final resolution = 10.8 Å
No Mask
Corrected
Masked
Phase Randomised
Fourier Shell Correlation
Resolution, Å
20.0
10.0
6.7
5.0
HINGE/ARM
GSFSC Resolution: 8.53Å
EMD-13894
No Mask (16Å)
Spherical (13Å)
Loose (8.8Å)
Tight (8.6Å)
Corrected (8.5Å)
HEAD-ONLY
GSFSC Resolution: 6.50Å
EMD-13893
No Mask (7.9Å)
Spherical (7.4Å)
Loose (6.9Å)
Tight (6.4Å)
Corrected (6.5Å)
DC
35Å
18Å
12Å
8.8Å
7Å
5.9Å
5Å
Resolution

Supplementary Figure 1

(a) Representative cryo-EM micrograph (b) Summary of image-processing workflow, used to determine the structure of the budding yeast Smc5/6 holo-complex. (c) Fourier Shell Correlation (FSC) curves estimating the average resolution of the indicated maps.

SUP. FIGURE 2

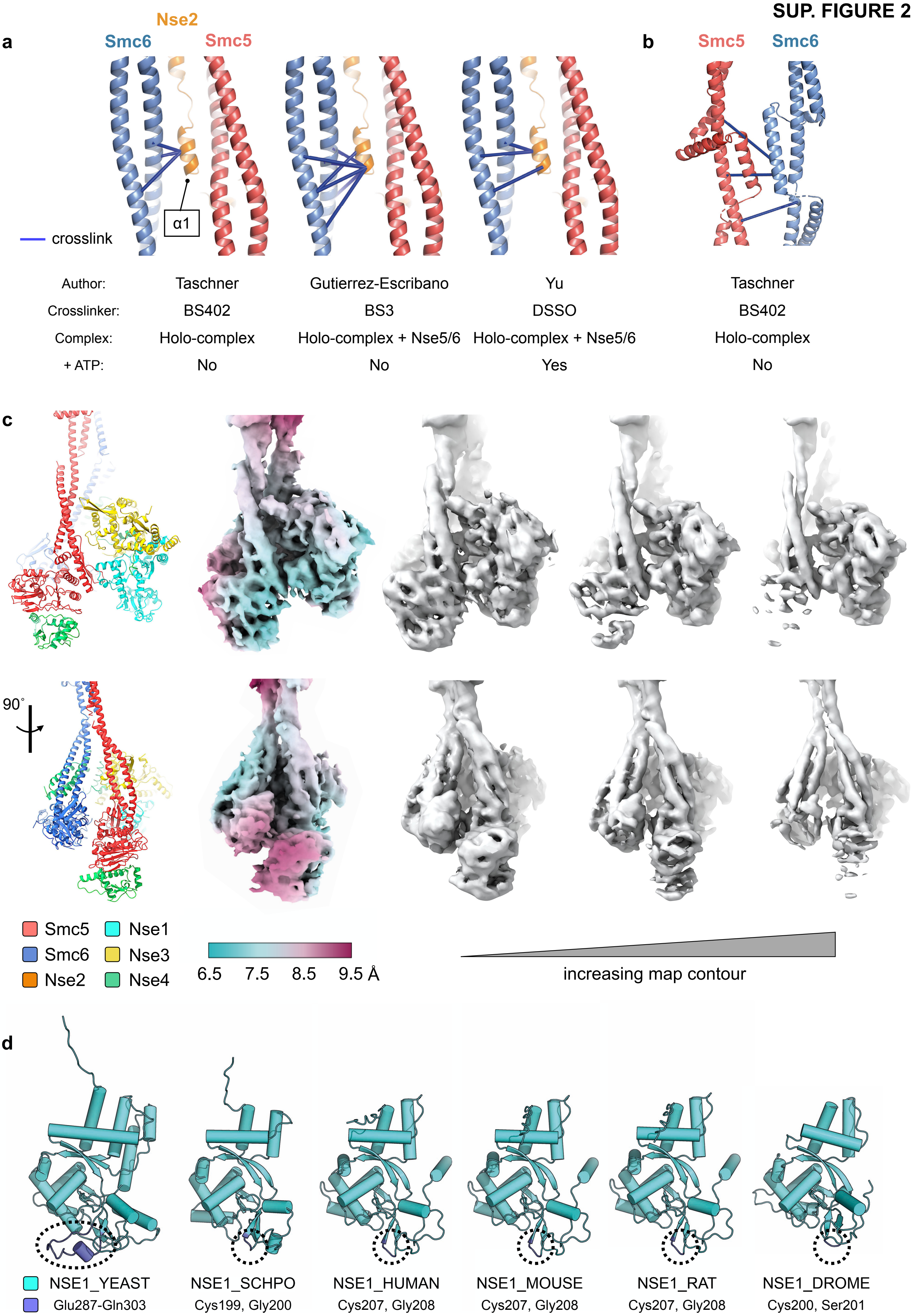

a
Nse2
Smc6
Smc5
α1
crosslink
Author: Taschner | Gutierrez-Escribano | Yu
Crosslinker: BS402 | BS3 | DSSO
Complex: Holo-complex | Holo-complex + Nse5/6 | Holo-complex + Nse5/6
+ ATP: No | No | Yes
b
Smc5
Smc6
Taschner
BS402
Holo-complex
No
c
90˚
Smc5
Smc6
Nse2
Nse1
Nse3
Nse4
6.5
7.5
8.5
9.5 Å
increasing map contour
d
NSE1_YEAST
Glu287-Gln303
NSE1_SCHPO
Cys199, Gly200
NSE1_HUMAN
Cys207, Gly208
NSE1_MOUSE
Cys207, Gly208
NSE1_RAT
Cys207, Gly208
NSE1_DROME
Cys200, Ser201

Supplementary Figure 2

(a) Selected cross-linking mass spectrometry (XL-MS) data taken from 3 separate studies, supporting interaction of the first helix of Nse2 (α2) with the arm of Smc5. Study, type of crosslinker used, complex composition and nucleotide status are summarised below the molecular graphics. Cross-links were visualised using the PyXLinkViewer plugin (ref. 1) for PyMOL (The PyMOL Molecular Graphics System, Version 2.3.2, Schrödinger, LLC). XL-MS data reported in (refs. 2, 3 and 4). (b) Selected XL-MS data taken from 2 supporting interaction of the two SMC 'joints' in the Smc5/6 holo-complex. (c) Different representations for the 'head-end' of the Smc5/6 complex. Sequentially from left to right: secondary structure molecular cartoon; estimate of local resolution as calculated by ResMap-1.1.4 (ref. 5) mapped onto the surface of the composite cryo-EM map; composite map shown at increasing contour levels. See associated keys for additional details. (d) AlphaFold predicts the presence of a budding yeast-specific loop insertion in Nse1 (amino acids 287-303, coloured dark blue).

SUP. FIGURE 3

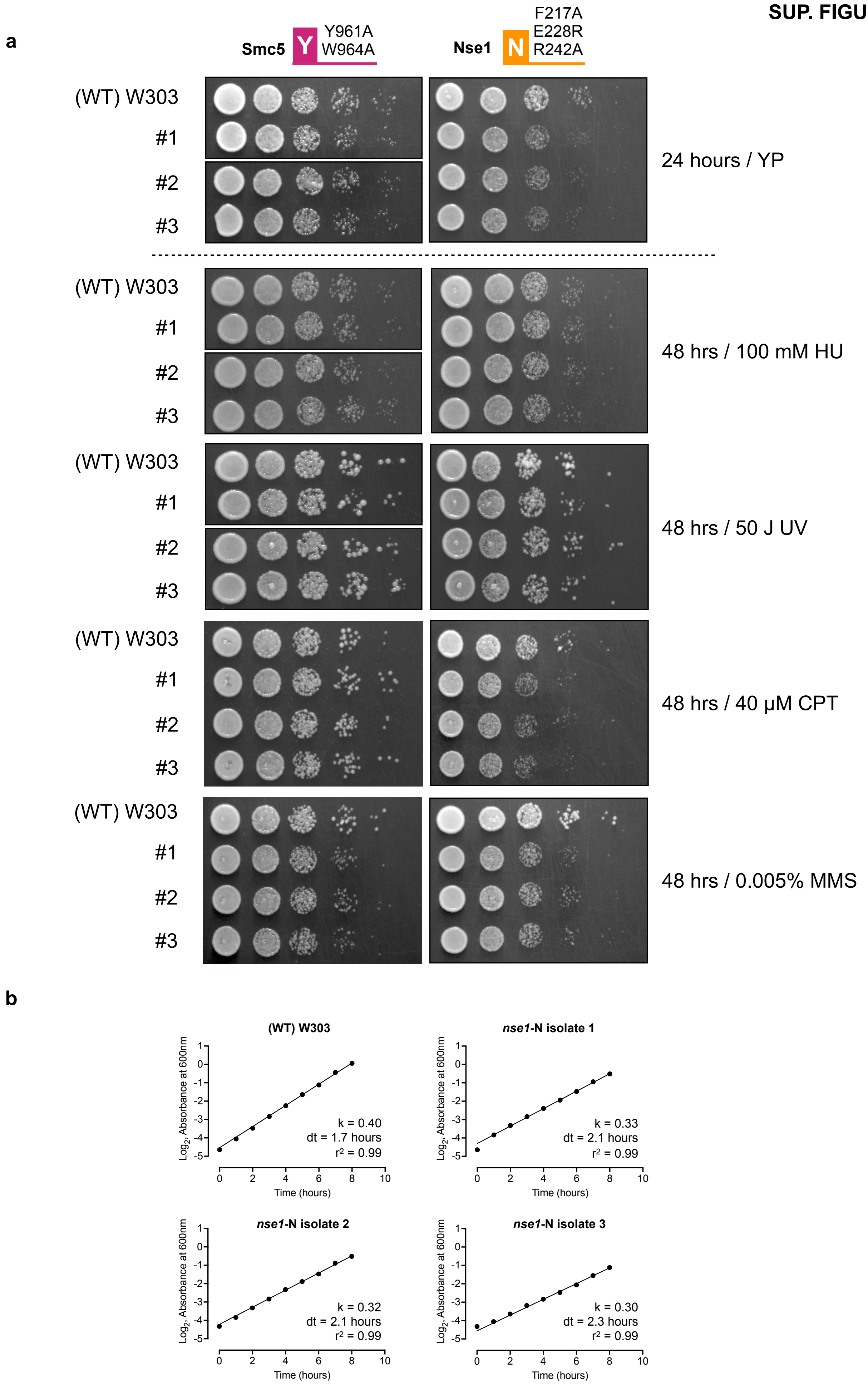
a
Smc5
Y
Y961A
W964A
Nse1
N
F217A
E228R
R242A
(WT) W303
#1
#2
#3
24 hours / YP
48 hrs / 100 mM HU
48 hrs / 50 J UV
48 hrs / 40 μM CPT
48 hrs / 0.005% MMS
b
(WT) W303
nse1-N isolate 1
nse1-N isolate 2
nse1-N isolate 3
Log2, Absorbance at 600nm
Time (hours)
k = 0.40
dt = 1.7 hours
r2 = 0.99
k = 0.33
dt = 2.1 hours
r2 = 0.99
k = 0.32
dt = 2.1 hours
r2 = 0.99
k = 0.30
dt = 2.3 hours
r2 = 0.99

Supplementary Figure 3

(a) Yeast strains carrying the Y-set of Smc5 mutations are not sensitive to a range of genotoxic agents and have no obvious growth defect. Those carrying the N-set of Nse1 mutations are mildly sensitive to treatment with camptothecin (CPT) or methyl methanesulphonate (MMS). Dose, type of treatment and duration of growth are as indicated; where HU = hydroxyurea, UV = ultraviolet light. The W303 strain is included as a wild-type (WT) control.

(b) Representative growth curves for the 3 isolates containing the N-set of mutations introduced into Nse1. The W303 strain is included as a wild-type (WT) control. Data are fitted to an Exponential (Malthusian) grown curve, with k defining a rate constant (slope), dt indicating the calculated doubling time, and r^2 a goodness of fit parameter.

SUP. FIGURE 4

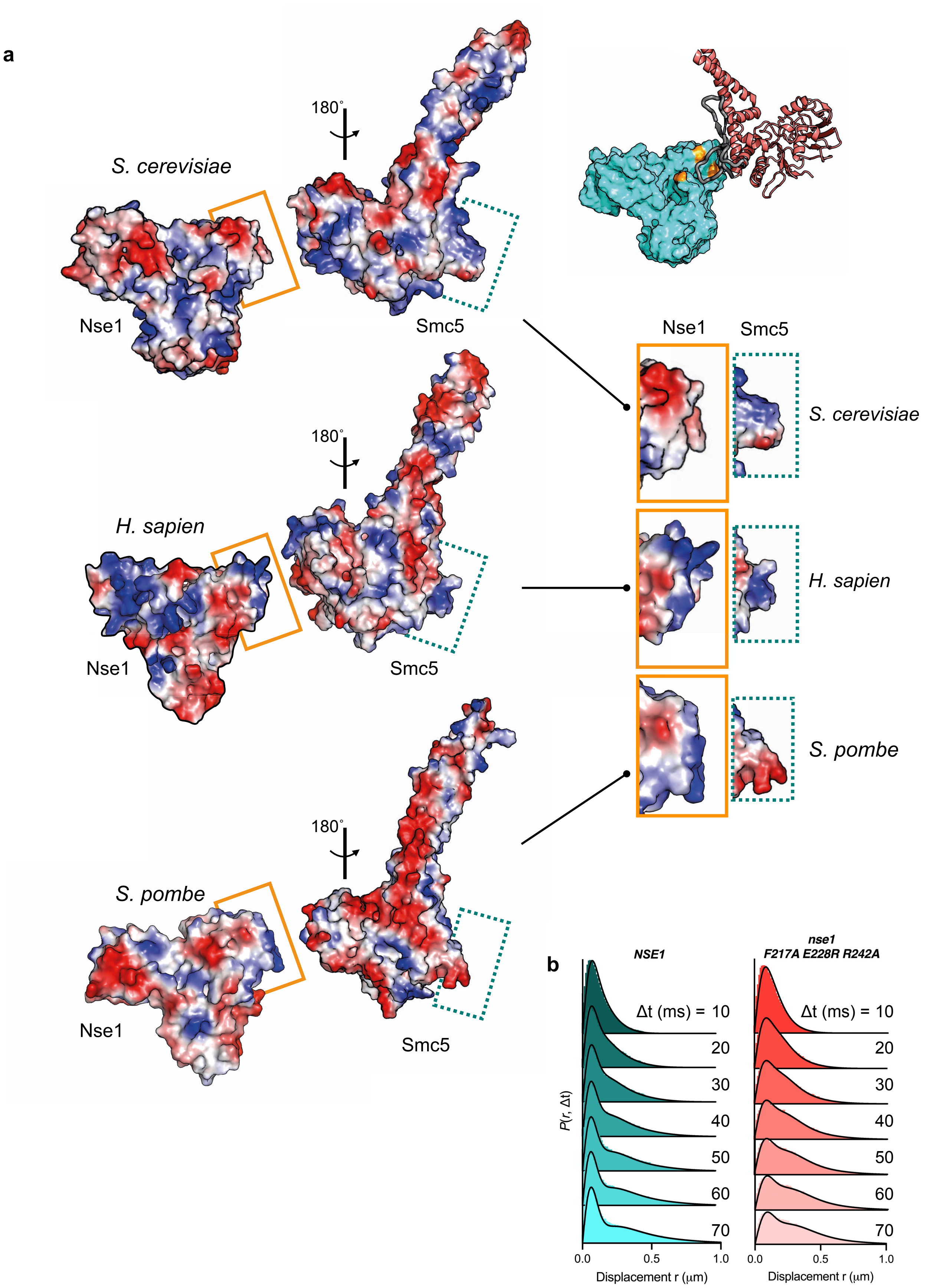
a
S. cerevisiae
180°
Nse1
Smc5
H. sapien
S. pombe
b
NSE1
nse1
F217A E228R R242A
Δt (ms) = 10
20
30
40
50
60
70
P(r, Δt)
0.0
0.5
1.0
Displacement r (μm)

Supplementary Figure 4

(a) Molecular surface representations for *S. cerevisiae*, *H. sapiens* and *S. pombe* Nse1 and corresponding head domain of Smc5, coloured by electrostatic potential (APBS plugin, PyMOL The surface of Nse1 and the conserved loop extending from Smc5 display a high degree of charge complimentary (visualisation aided by rotation of the head domain through 180°). The interacting regions, as bounded by the drawn rectangles, are shown in an expanded view on the right. AlphaFold accession codes for each model: AF-Q07913-F1 (NSE1_YEAST), AF-Q08204-F1 (SMC5_YEAST), AF-Q8WV22-F1 (NSE1_HUMAN), AF-Q8IY18-F1 (SMC5_HUMAN), AF-Q53EK2-F1 (NSE1_SCHPO), AF-O13710-F1 (SMC5_SCHPO). (a, inset) Molecular secondary structure cartoon highlighting the position of the mutated amino acid residues in Nse1 (carbon atoms coloured in orange and shown in 'stick' representation) relative to that of the Smc5 'W-loop' (coloured in dark grey, with the side chain of Leu978 shown in 'stick' representation). Please also see associated key for additional details. (b) Probability density function plots, with fitting (Spot-On, ref. 6; solid line) for Nse4–Halo-JFX646 single-molecule displacements over the first 8 frames (10ms-70ms), in *NSE1* and *nse1-F217A E228R R242A* genetic backgrounds. Displacements were calculated from three pooled independent experiments, each containing three technical repeats.

a

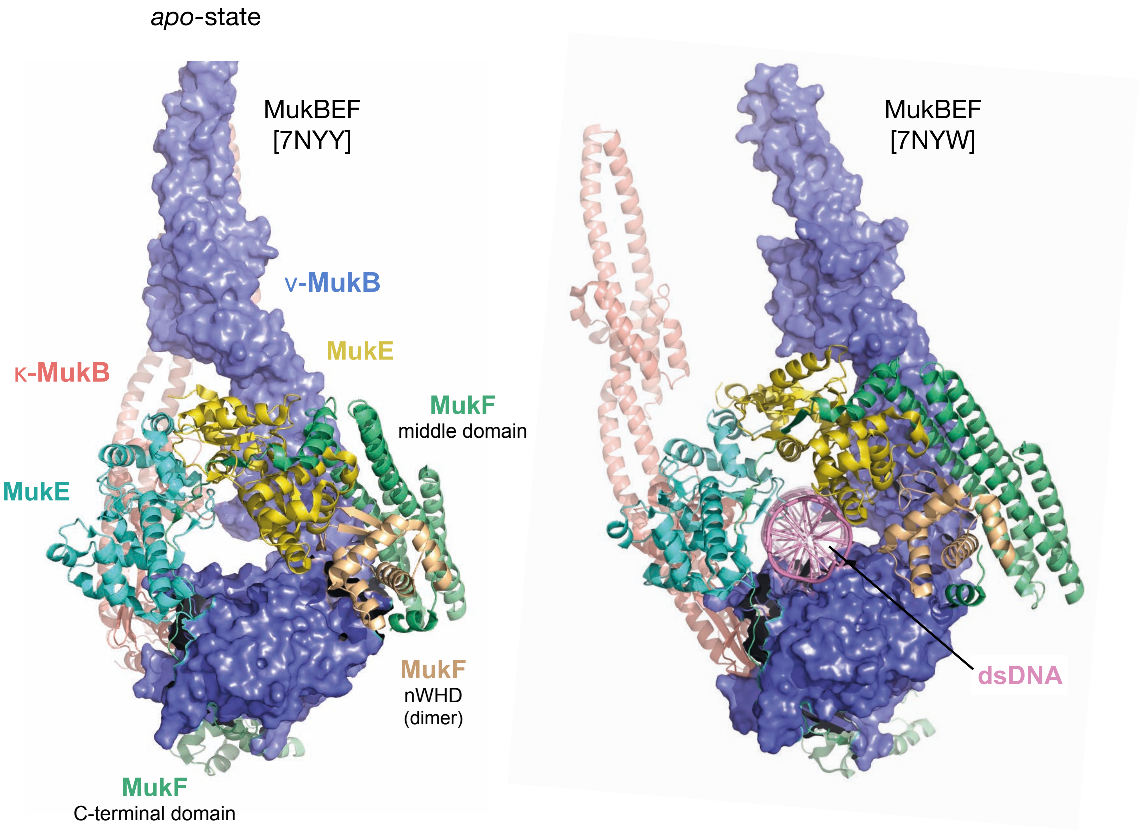

apo-state
MukBEF
[7NYY]
ν-MukB
MukE
κ-MukB
MukF
middle domain
MukE
MukF
nWHD
(dimer)
MukF
C-terminal domain
MukBEF
[7NYW]
dsDNA

b

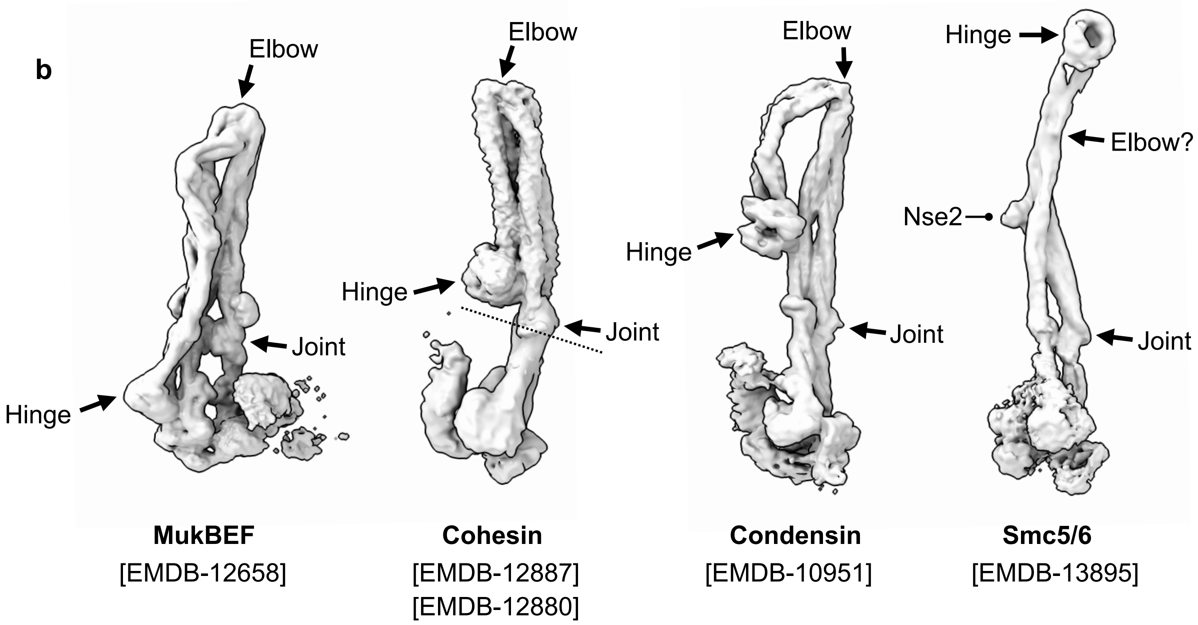

Elbow
Joint
Hinge
Elbow
Hinge
Joint
Elbow
Hinge
Joint
Hinge
Elbow?
Nse2
Joint
MukBEF
[EMDB-12658]
Cohesin
[EMDB-12887]
[EMDB-12880]
Condensin
[EMDB-10951]
Smc5/6
[EMDB-13895]

Supplementary Figure 5

(a) Side-by-side visualisation of the reported *apo* (left) and fully 'engaged' / DNA-bound states (right) of MukBEF (ref. 7), positioned relative to each other using the head domain / arm of the ν-MukB moiety as a fixed reference (shown as a molecular surface); ν-MukB/MukE appears to behave largely as a rigid body, already 'primed' to interact and accept a DNA duplex, potentially obviating the need for a large conformation change driven by a 'flip-flop' type mechanism. (b) Representative cryo-EM map for MukBEF (ref. 7) shown alongside those for the reported *apo*-states of budding yeast cohesin (ref. 8), condensin (ref. 9) and Smc5/6 (this paper). EMDB accession codes are as indicated.

References

1. Schiffrin, B., Radford, S.E., Brockwell, D.J. and Calabrese, A.N. (2020) PyXlinkViewer: A flexible tool for visualization of protein chemical crosslinking data within the PyMOL molecular graphics system. *Protein Sci*, **29**, 1851-1857.
2. Taschner, M., Basquin, J., Steigenberger, B., Schafer, I.B., Soh, Y.M., Basquin, C., Lorentzen, E., Raschle, M., Scheltema, R.A. and Gruber, S. (2021) Nse5/6 inhibits the Smc5/6 ATPase and modulates DNA substrate binding. *EMBO J*, e107807.
3. Gutierrez-Escribano, P., Hormeno, S., Madariaga-Marcos, J., Sole-Soler, R., O'Reilly, F.J., Morris, K., Aicart-Ramos, C., Aramayo, R., Montoya, A., Kramer, H. *et al.* (2020) Purified Smc5/6 Complex Exhibits DNA Substrate Recognition and Compaction. *Mol Cell*, **80**, 1039-1054 e1036.
4. Yu, Y., Li, S., Ser, Z., Sanyal, T., Choi, K., Wan, B., Kuang, H., Sali, A., Kentsis, A., Patel, D.J. *et al.* (2021) Integrative analysis reveals unique structural and functional features of the Smc5/6 complex. *Proc Natl Acad Sci U S A*, **118**.
5. Kucukelbir, A., Sigworth, F.J. and Tagare, H.D. (2014) Quantifying the local resolution of cryo-EM density maps. *Nat Methods*, **11**, 63-65.
6. Hansen, A.S., Woringer, M., Grimm, J.B., Lavis, L.D., Tjian, R. and Darzacq, X. (2018) Robust model-based analysis of single-particle tracking experiments with Spot-On. *Elife*, **7**.
7. Burmann, F., Funke, L.F.H., Chin, J.W. and Lowe, J. (2021) Cryo-EM structure of MukBEF reveals DNA loop entrapment at chromosomal unloading sites. *Mol Cell*, **81**, 4891-4906 e4898.
8. Petela, N.J., Gonzalez Llamazares, A., Dixon, S., Hu, B., Lee, B.G., Metson, J., Seo, H., Ferrer-Harding, A., Voulgaris, M., Gligoris, T. *et al.* (2021) Folding of cohesin's coiled coil is important for Scc2/4-induced association with chromosomes. *Elife*, **10**.
9. Lee, B.G., Merkel, F., Allegretti, M., Hassler, M., Cawood, C., Lecomte, L., O'Reilly, F.J., Sinn, L.R., Gutierrez-Escribano, P., Kschonsak, M. *et al.* (2020) Cryo-EM structures of holo condensin reveal a subunit flip-flop mechanism. *Nat Struct Mol Biol*, **27**, 743-751.
